# Nerve decompressive surgery of the lower limbs for diabetic peripheral neuropathy: a systematic review and meta-analysis

**DOI:** 10.3389/fpain.2026.1879398

**Published:** 2026-07-30

**Authors:** Yufen Zhao, Jiaying Chen, Zirui Li, Yanji Zhang, Mingyuan Tang, Yunxue Zhong, Yang Jian, Chengliang Deng, Zairong Wei

**Affiliations:** 1 Department of Burns and Plastic Surgery, Affiliated Hospital of Zunyi Medical University, Zunyi, Guizhou, China; 2 Department of Lymphatic Surgery, Bishan Hospital of Chongqing Medical University, Chongqing, China; 3 The Collaborative Innovation Center of Tissue Damage Repair and Regeneration Medicine of Zunyi Medical University, Zunyi, Guizhou, China

**Keywords:** diabetic peripheral neuropathy, pain, nerve decompressive surgery, nerve conduction velocity, systematic review

## Abstract

**Objective:**

There is no consensus on the efficacy and safety of nerve decompressive surgery of lower limbs (NDSLL) for diabetic peripheral neuropathy (DPN). This systematic review aims to evaluate the efficacy and safety of NDSLL compared with no surgery in DPN by analyzing relevant evidence from randomized controlled trials (RCTs).

**Methods:**

Using PubMed, Embase, Cochrane Library, ClinicalTrials.org and Chictr.org.cn, a systemic literature review was performed in accordance with the Preferred Reporting Items for Systemic Reviews and Meta-Analyses statement (CRD42021295459).

**Results:**

Screening filtered 793 studies to 7 trials containing 207 patients. Only two of the eight registered RCTs have been published (25% publication rate), indicating substantial publication bias. Risk of bias was high across most trials, with particular concerns regarding: lack of participant and personnel blinding; absence of sham surgery controls in 6/7 trials; incomplete outcome data; and selective reporting. These methodological flaws likely contribute to overestimation of treatment effects. Compared to no surgery, NDSLL was associated with VAS reduction at postoperative month (POM) 6 (WMD: −2.77, 95% CI: −3.84 to −1.69) and POM 12 (WMD: −2.41, 95% CI: −4.52 to −0.29). However, wide confidence intervals—particularly at 12 months—and high bias risk limit confidence in these estimates. No RCT evidence supported improvement in quality of life, nerve conduction velocity, balance, ulcer healing, or sensation. Among 113 NDSLL patients, 27 (23.9%) experienced wound-related complications (infection, dehiscence, edema, hematoma); no complications were reported in no-surgery groups.

**Conclusions:**

NDSLL may offer pain relief in selected DPN patients, but the current evidence is derived from a small number of high-bias RCTs with substantial publication bias, considerable methodological heterogeneity, and a notable complication rate. The true treatment effect may be substantially smaller than pooled estimates suggest. The absence of demonstrable benefit on quality of life, nerve function, balance, ulcer healing, or sensation constrains clinical utility. NDSLL should remain an investigational procedure pending adequately powered, sham-controlled, multicenter RCTs with standardized outcome measures. More high-quality research is urgently needed before NDSLL can be recommended as standard treatment.

**Systematic Review Registration:**

https://www.crd.york.ac.uk/PROSPERO/view/CRD42021295459, PROSPERO CRD42021295459.

## Introduction

Diabetic peripheral neuropathy (DPN) is a common complication of diabetes that significantly reduces patients’ quality of life (QOL) by increasing fall risk, restricting activities of daily living, and causing pain and depression (1, 2). Approximately 50% of adults with diabetes will experience DPN during their lifetime (3). DPN imposes a substantial financial burden (3, 4). In the USA, the total annual cost of DPN is estimated at $10.9 billion, with up to 27% of the direct medical costs associated with diabetes (5). Furthermore, patients with DPN show high morbidity of foot ulcer and amputation (3, 4).

DPN is irreversible, and currently, no effective treatment methods exist. Glycemic control, altering metabolic abnormalities, and analgesics have been used to treat DPN. Although improved glycemic control can prevent the occurrence and progression of DPN (6), clinical evidence for drugs that regulate metabolism is limited (7). Pain medications can only reduce neuropathic symptoms but cannot prevent the underlying nerve pathology or progression of neuropathy (8). New therapies, such as electrical stimulation, low-intensity laser, and stem cells, have been developed to treat DPN. However, the current evidence is insufficient to confirm their benefits or exclude their drawbacks (9). A 1992 prospective study by Dellon et al. (10) on nerve decompression surgery found that neurological symptoms significantly improved in 60 adults with DPN (51 upper limbs and 31 lower limbs, involving 154 peripheral nerves) after a mean follow-up of 30 months after surgery. Subsequently, multiple studies from different centers involving many participants have been published, indicating that nerve decompressive surgery for DPN is widely used in many therapeutic centers (11). However, the effect and safety of NDSLL, both in general and specifically for DPN, remain controversial (11–13). In 2006, the Therapeutics and Technology Assessment Subcommittee of the American Academy of Neurology published a practice advisory stating that “this treatment alternative should be considered unproven.” (14). A systematic review in 2008 also reached a similar conclusion (15). However, two other systematic reviews have shown that nerve decompressive surgery effectively improves neurologic symptoms and restores sensory function in patients with DPN (16, 17).

Consensus has not yet been reached on the efficacy and safety of nerve decompressive surgery of the lower limbs (NDSLL) for DPN (11). Therefore, this systematic review and meta-analysis evaluates the efficacy of NDSLL vs. no surgery in relieving symptoms and restoring sensation in DPN patients, assesses associated complications, and offers evidence-based recommendations for its clinical use. Given the ongoing controversy surrounding NDSLL since its introduction in 1992, with some systematic reviews suggesting benefit while official practice advisories consider it unproven, we conducted this systematic review and meta-analysis to critically appraise the highest level of available evidence (RCTs). Our objectives were to: quantitatively synthesize pain outcomes from all available RCTs; systematically assess the quality and bias profile of the evidence; evaluate secondary outcomes including QOL, NCV, sensory recovery, and complications; and identify critical knowledge gaps that must be addressed before NDSLL can be endorsed in clinical practice. By explicitly focusing on RCTs and employing rigorous methodological standards, we aimed to provide clinicians, researchers, and guideline developers with a transparent and balanced assessment of what is—and what is not—currently known about NDSLL for DPN.

## Methods

This systematic review and meta-analysis followed preferred reporting items for systematic reviews and meta-analysis guidelines and was registered with PROSPERO (CRD42021295459) (18). The search strategies for the databases are provided in Supplementary Appendix 1. The databases included PubMed, Embase, the Cochrane Library, with searches conducted from inception through April 17, 2024. ClinicalTrials.org and Chictr.org.cn were searched for trial registrations from inception through April 28, 2024. We supplemented our search results by examining the references of four other relevant systematic reviews (15–17, 19).

RCTs analyzing NDSLL vs. no surgery (control group) were included. NDSLL included the common peroneal nerve at the fibular neck, the deep peroneal nerve on the foot dorsum, and the tibial, medial, and lateral plantar nerves at the medial malleolus. The inclusion and exclusion criteria are shown in Supplementary Appendix 2. The primary outcome was the change in pain measured by the visual analog scale (VAS) or other scales at the foot between the baseline and a follow-up period of over six months. Secondary outcomes included any validated assessments of nerve function, QOL, surgical complications, and diabetic foot ulcers between baseline and a more than three-month follow-up period.

Screening, extraction, and certainty assessment were performed independently by two co-authors. Conflicts were solved by mutually reviewing the article. The following data were extracted from each included study: the first author's name, geographic region, population source, year of publication, demographic information, number of patients, length of follow-up, outcomes including VAS, Likert scale or the McGill Pain Questionnaire, neuroelectrophysiology improvements, two-point discriminator (2-PD), surgical complications, complications of DPN, QOL, and neurological imaging changes. Mean with confidence interval (CI), mean with standard error of the mean, or mean with *p*-values were converted to mean and standard deviation (SD). Studies providing the median with an interquartile range (IQR) or range were converted to the mean with SD as appropriate (20–23).

According to the Cochrane risk of bias (ROB) assessment tool, ROB assessment for RCTs was conducted independently by two co-authors. An ROB table was constructed for all included RCTs, containing random sequence generation, allocation concealment, blinding of participants, blinding of outcome assessment, incomplete outcome data, selective reporting, and other sources of bias.

We acknowledge that the number of included RCTs is limited. However, our comprehensive search strategy—covering five major databases (PubMed, Embase, Cochrane Library, ClinicalTrials.gov, and Chictr.org.cn) from inception through April 2024, supplemented by manual screening of reference lists from relevant systematic reviews—ensures that we have captured all currently available RCTs evidence on this topic. Thus, the small number of included studies reflects the current state of the evidence base rather than a limitation of our retrieval process.

Review Manager (RevMan 5.3) software was performed to compare the outcomes between the NDSLL and no surgery groups. Heterogeneous data between studies were indicated by *p* ≤ 0.10 or *I*^2^ ≥ 50%; otherwise, the data were homogeneous. Random-effect and fixed-effect models were used to analyze heterogeneous and homogeneous data, respectively. Pooled weighted mean difference (WMD) and proportions with 95% CI and *p*-values were calculated. The test of overall effect with *p* < 0.05 was considered statistically significant. When enough studies were included, the funnel plot was delineated, and the publication bias was evaluated.

## Results

**Figure 1:**
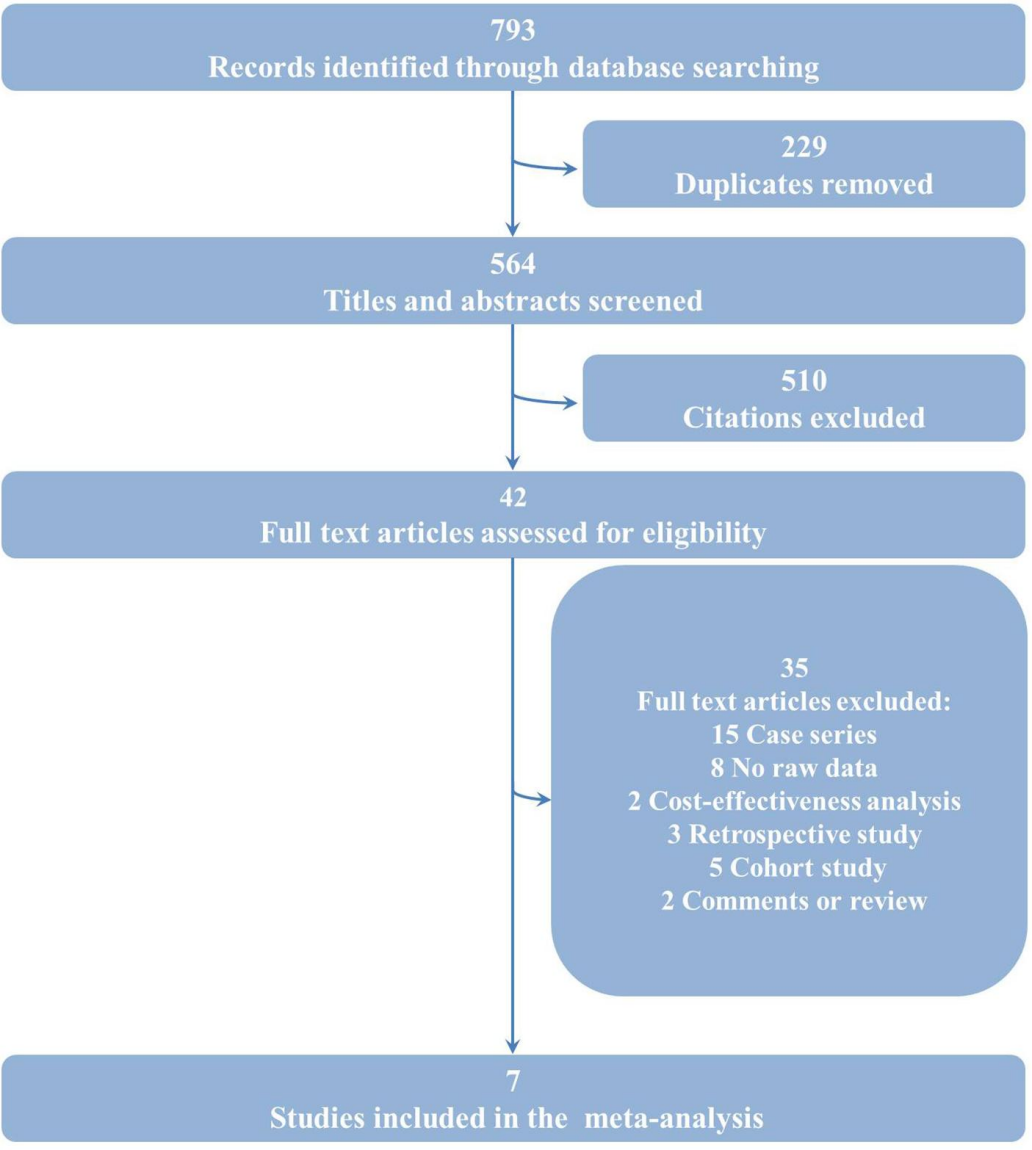
Study selection flowchart.

Of 793 studies, 7 RCTs met the inclusion criteria (Figure 1), Most RCTs (85.71%) are in place in developed countries (Supplementary Appendix 5) (23–29). A search on ClinicalTrials.gov and Chictr.org.cn revealed 8 protocols; 2 completed and published (24, 26). All trials meeting the inclusion criteria were captured or unavailable (Supplementary Appendix 3). We integrated the articles on the lower extremity nerve entrapment study (LENS) published by Macare van Maurik et al. (23, 27–29).

**Figure 2:**
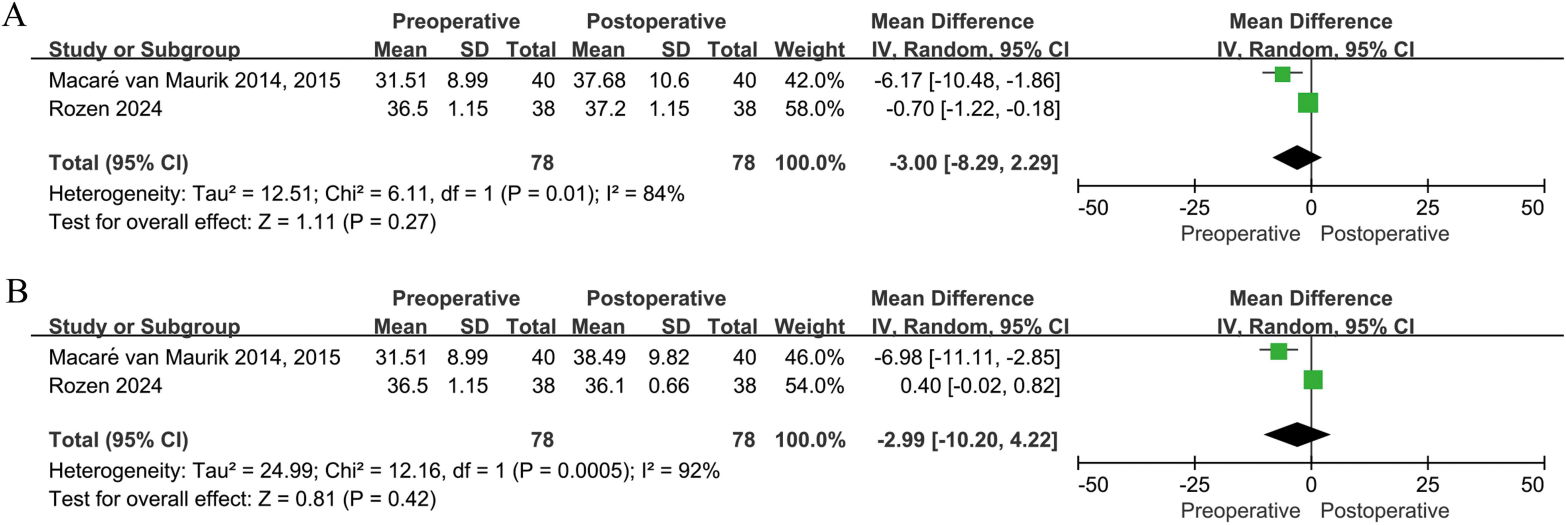
Summaries of risk of bias. **(A)** Detailed risk of bias assessments. **(B)** Risk of bias graph.

Study characteristics are reported (Table 1, Supplementary Appendices 4 and 5) (25). These studies, published from 2014 to 2024 (Supplementary Appendix 5), include 207 patients. The NDSLL group included 59 males and 54 females, and no surgery group included 92 males and 77 females. One RCT reported sham operation (the contralateral leg of the NDSLL) involving 19 males and 21 females (24), One study used self-control (left and right leg) to include 22 males and 16 females (Supplementary Appendix 5) (23, 27–29). Type 2 diabetes (93.72%) was the most common (Table 1). Two studies reported BMI (Supplementary Appendix 5) (23, 24, 27–29). Only one RCT reported the duration of pain (25) and one RCT reported DPN duration (Supplementary Appendix 5) (24). Most RCTs (85.71%) are in place in developed countries and most RCTs (85.71%) reported HbA1c (Supplementary Appendix 5) (23, 24, 26–29). Positive Tinel sign was often used as an indicator for NDSLL (Supplementary Appendix 4) (23–25, 27–29). Sham operation was not done in most trials (85.71%) (Table 1). All RCTs included in this review were judged to be at high ROB for incomplete outcome data and selective reporting. Most RCTs (75%) mentioned a low ROB for randomization. Most RCTs (75%) mentioned a high ROB for blinding participants and personnel, and other sources of bias. Half of the RCTs had a high ROB for blinding of outcome assessment and allocation concealment (Figures 2A,B). Of the eight RCTs prospectively registered on ClinicalTrials.gov and Chictr.org.cn for this indication, only two (25%) have been published in peer-reviewed journals as of April 2024 (Supplementary Appendix 3). The remaining six registered trials remain unpublished, with no accessible results in public registries or peer-reviewed literature. This 75% non-publication rate raises serious concerns about selective reporting bias: trials with positive or statistically significant results are substantially more likely to be published than those with null or negative findings. Consequently, the published literature likely represents a biased subset of all conducted RCTs, and our pooled estimates—derived exclusively from published studies—may systematically overestimate the true efficacy of NDSLL.

**Table 1:** Characteristics of included studies.

Study ID	Number of patients	Design	Type 2 diabetes, n (%)	Intervention (Sample)	Control (Sample)	Age (means ± SD)		Diabetes duration (years)		Pain scale used	QOL	Neurological assessment	Follow up (months)
						Intervention	Control	Intervention	Control				
Rozen 2024	78	Single-center, patient- and rater-blinded, observation- and same-patient sham surgery-controlled RCT	74	NDSLL (37)	A: sham (37). B: No surgery (34)	51.73 ± 13.30	A: 51.73 ± 13.30.	Unknown	Unknown	Likert scale	SF-36	NR	56
							B: 54.62 ± 9.86.						
Ma 2023	69	Single-center, single-blinded, three-arm RCT	69	NDSLL (23)	A: No surgery (23). B: NDSLL + nerve conduit (23)	66.8 ± 9.9	A: 62.2 ± 11.5.	9.74 ± 4.3	A: 10.2 ± 5.0	VAS	NR	TCSS score; NCV; 2-PD	6
							B: 62.4 ± 10.4		B: 11.35 ± 4.7				
Best 2019	22	Single-center, single-blinded, parallel-group RCT	21	NDSLL (12)	No surgery (10)	64 ± 6.38	68 ± 7.13	11.63 ± 9.63	7.14 ± 3.08	VAS	NeuroQoL	PSSD; TCSS score	12
Macaré van Maurik 2014, 2015	38	Single-center, unblinded, within-patient comparison RCT	30	NDSLL (35)	No surgery (35)	61.7 ± 10.2	61.7 ± 10.2	17.6 ± 12.2	17.6 ± 12.2	VAS	SF-36; EQ-5D questionnaires	NCV; sway measurements; SWMT score; 2-PD	12

2-PD: two-point discriminator; EQ-5D, EuroQol five dimensions; NDSLL, nerve decompressive surgery of lower limbs; NR, not reported; NCV, nerve conduction velocity; NeuroQoL, neuropathy-specific quality of life; PSSD, pressure-specified sensory device; QOL, quality of life; RTC, randomized clinical trial; SF-36, the 36-item shot-form health status survey; SWMT, semmes weinstein monofilament testing; TCSS, toronto clinical scoring system score; VAS, visual analogue scale.

**Figure 3:**
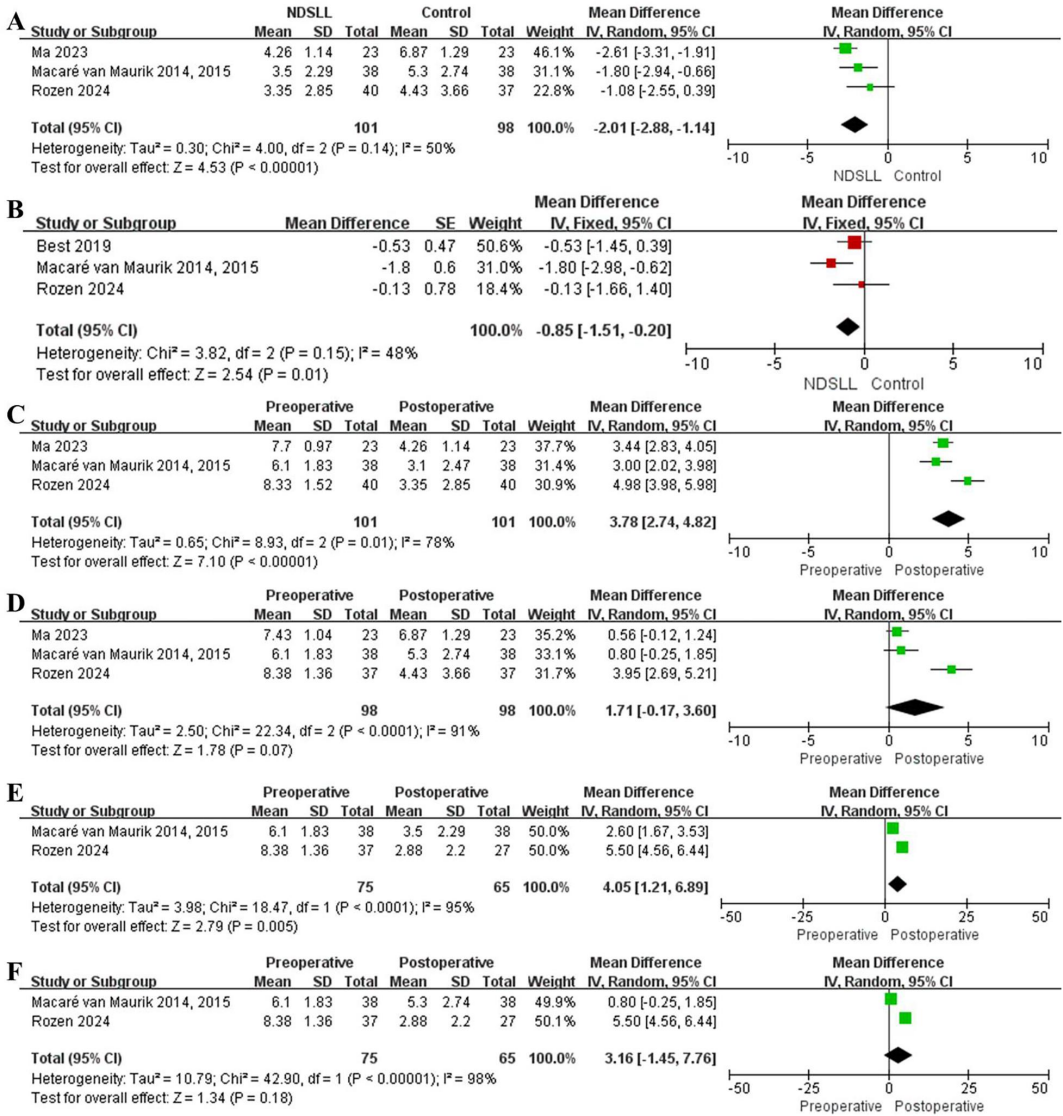
Forest plots of pooled analysis of VAS. **(A)** Pooled analysis of NDSLL vs. no-surgery VAS at 6 months after surgery. **(B)** Pooled analysis of NDSLL vs. no-surgery VAS at 12 months after surgery. **(C)** Pooled analysis of preoperative vs. postoperative VAS in the NDSLL group at 6 months after surgery. **(D)** Pooled analysis of preoperative vs. postoperative VAS in the no-surgery group at 6 months after surgery. **(E)** Pooled analysis of preoperative vs. postoperative VAS in the NDSLL group at 12 months after surgery. **(F)** Pooled analysis of preoperative vs. postoperative VAS in the control group at 12 months after surgery. NDSLL, nerve decompressive surgery of lower limbs; VAS, visual analog scale.

Three RCTs reported VAS outcomes (23, 25, 26, 28), and one RCT reported a Likert scale (24). Therefore, we analyzed the VAS results. Three studies reported VAS outcomes at six months (23–25, 28). A pooled analysis of NDSLL vs. no surgery group in the VAS showed a significant improvement at postoperative months (POM) 6 (WMD: −2.77, 95% CI: −3.84 to −1.69, *p* < 0.00001) (Figure 3A). Three RCTs reported differences in the VAS at POM 12 between the NDSLL and control groups (23, 24, 26, 28), and there was significant difference in VAS (WMD: −2.41, 95% CI: −4.52 to −0.29, *p* = 0.01) (Figure 3B). To contextualize the clinical relevance of these reductions, the minimal clinically important difference (MCID) for VAS in chronic neuropathic pain is generally accepted as a reduction of 1.5–2.0 points on a 0–10 scale, or approximately a 30% decrease from baseline. While our pooled WMD at 6 months (−2.77) exceeds this threshold on average, the relatively wide confidence intervals, particularly at 12 months (95% CI: −4.52 to −0.29, lower bound approaching zero), indicate that the true effect may fall below the MCID in a substantial proportion of patients. This imprecision, combined with the high risk of bias and publication bias, warrants considerable caution in interpreting these estimates as evidence of clinically meaningful benefit.

Three RCTs reported preoperative and postoperative changes in the VAS (23, 24–26, 28). Compared to before surgery, VAS showed a significant improvement in the NDSLL group (WMD at POM 6: 3.78, 95% CI: 2.74–4.82, *p* < 0.00001; WMD at POM 12: 3.97, 95% CI: 1.31–6.64, *p* = 0.003) (Figures 3C,E). In contrast, the no surgery group showed only a slight improvement at POM 6 (WMD: 0.64, 95% CI: 0.14–1.14, *p* = 0.01) (Figure 3D), with no significant improvement in pain at POM 12 (WMD: 0.72, 95% CI: 0.07–1.37, *p* = 0.03) (Figure 3F).

**Figure 4:**
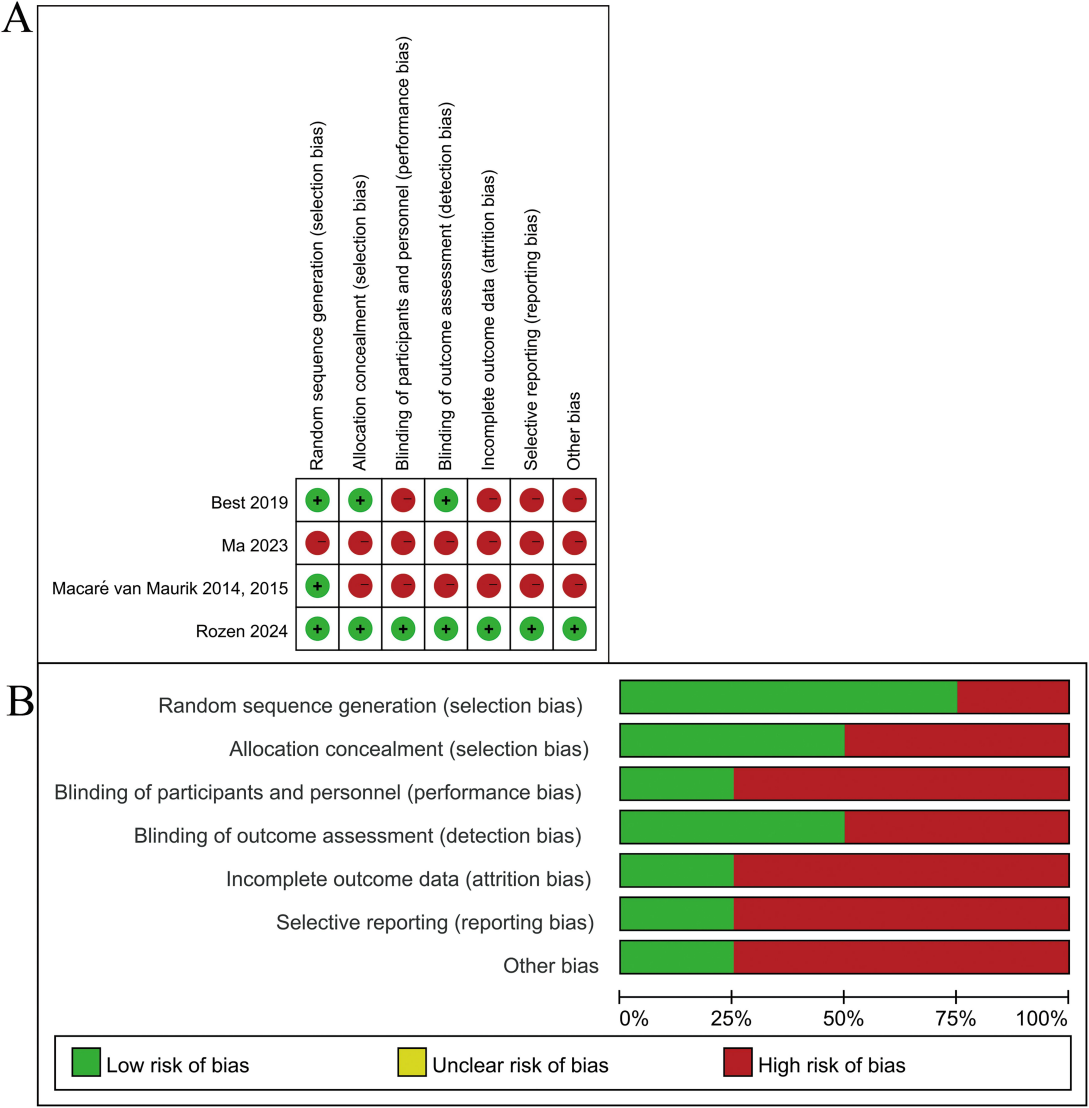
Pooled analysis of physical component summary score. **(A)** Pooled analysis of QOL in the NDSLL group at 6 months after surgery. **(B)** Pooled analysis of QOL in the NDSLL group at 12 months after surgery. NDSLL, nerve decompressive surgery of lower limbs; QOL, quality of life.

The difference of QOL assessment was high because of the inconsistent QOL assessment tools used in the studies. The EuroQol five-dimension (EQ-5D) questionnaires (28), and QOL in neurological disorders (NeuroQoL) (26) were each reported in one RCT. Two RCTs reported the 36-item short-form survey (SF-36) (24, 28), and the physical component summary score was pooled analysis. There was no significant improvement in QOL in the NDSLL group (WMD at POM 6: −3.00, 95% CI: −8.29 to 2.29, *p* = 0.27; WMD at POM 12: −2.99, 95% CI: −10.20 to 4.22, *p* = 0.42) (Figures 4A,B).

**Figure 5:**
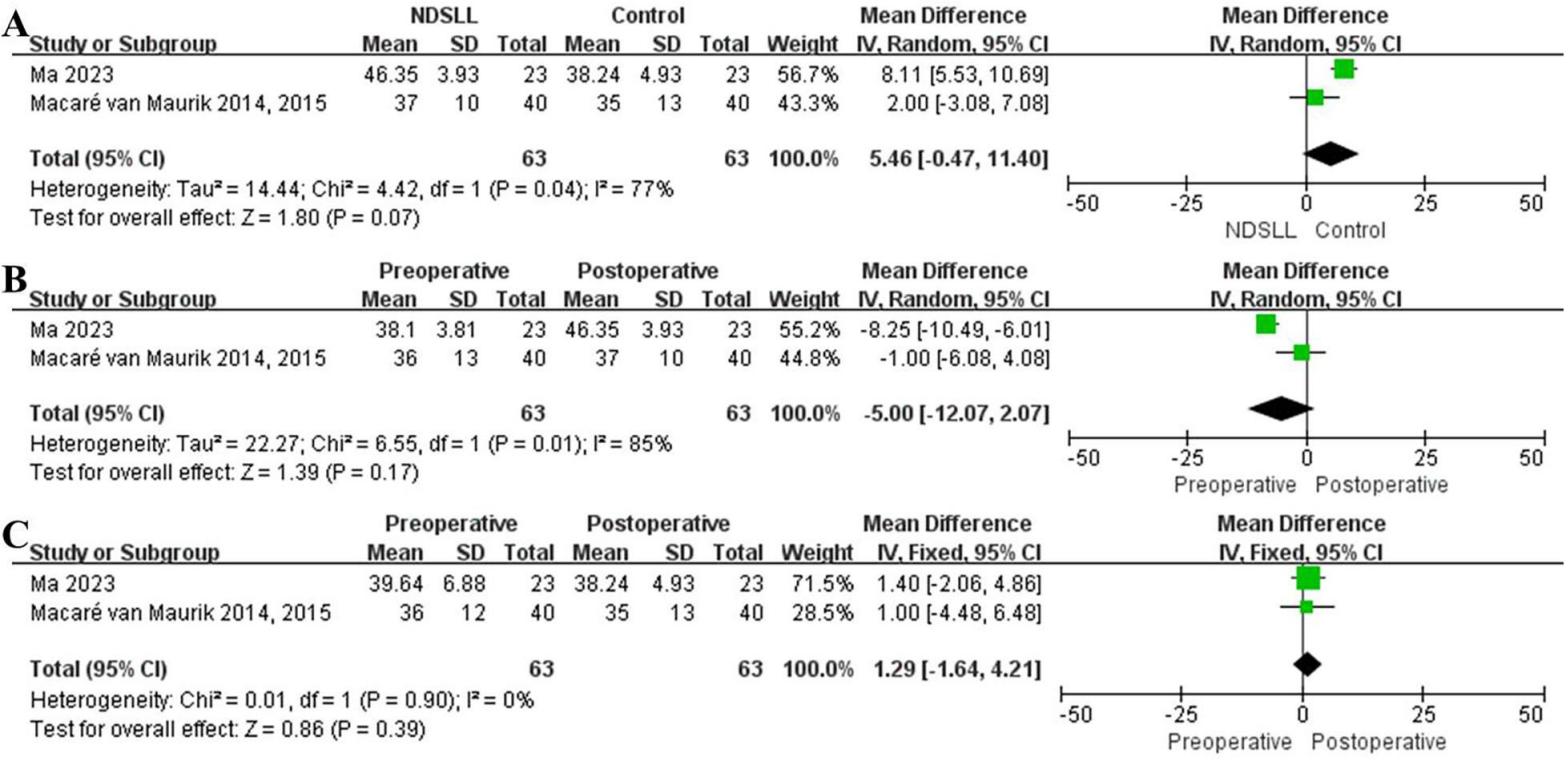
Pooled analysis of posterior tibial NCV. **(A)** Pooled analysis of NDSLL vs. no-surgery in posterior tibial NCV. **(B)** Pooled analysis of preoperative vs. postoperative posterior tibial NCV in the NDSLL group. **(C)** Pooled analysis of preoperative vs. postoperative posterior tibial NCV in the no-surgery group. NCV, nerve conduction velocity; NDSLL, nerve decompressive surgery of lower limbs.

Two RCTs reported nerve conduction velocity (NCV) outcomes (25, 29). For posterior tibial NCV, pooled analysis of NDSLL vs. no surgery group in the posterior tibial NCV showed no significant improvement after surgery (WMD: 5.46, 95% CI: −0.47 to 11.40, *p* = 0.07) (Figure 5A). In the NDSLL group, there was no significant improvement in the posterior tibial NCV after surgery (WMD: −5.00, 95% CI: −12.07 to 2.07, *p* = 0.17) (Figure 5B). There was also no significant improvement in the posterior tibial NCV in no surgery group (WMD: 1.29, 95% CI: −1.64 to 4.21, *p* = 0.39) (Figure 5C). For common peroneal NCV, NDSLL showed no significant improvement in common peroneal NCV compared with no surgery group (WMD: 4.18, 95% CI: −7.59 to 15.96, *p* = 0.49) (Supplementary Appendix 6A). There was no significant improvement in common peroneal NCV before and after operation (WMD in the NDSLL group: −5.54, 95% CI: −16.17 to 5.09, *p* = 0.31; WMD in the no surgery group: 0.39, 95% CI: −2.59 to 3.37, *p* = 0.80) (Supplementary Appendices 6 B and C).

Three RCTs reported adverse events (23, 24, 26–29). Of the 113 patients in the NDSLL group, 27 had treatment-related adverse events. These included 10 cases of infection, 12 cases of wound dehiscence, 4 cases of edema, and 1 of hematoma. No treatment-related adverse events were reported in the no-surgery group. Two RCTs reported 2-PD outcomes (23, 25) and Toronto clinical scoring system (TCSS) score (25, 26), but a meta-analysis of these outcomes was not feasible due to incomplete reporting, one study reported only median values without dispersion measures, while another presented results in a format that could not be converted to mean and standard deviation. This inability to pool these secondary outcomes is itself a significant limitation, the pattern of missing data may reflect selective reporting, where studies that did not find improvements in these measures may have been less likely to report them fully. Therefore, while individual studies suggested potential sensory improvements, the absence of pooled evidence prevents us from drawing robust conclusions about sensory recovery, and claims regarding neurological benefit remain unsubstantiated by meta-analyzable RCT data. Semmes-Weinstein monofilament testing score (23), sway measurements (27), and the pressure-specified sensory device (26) were each reported in one RCT. No RCTs reported imaging changes or examined the relationship between NDSLL and diabetic foot ulcer healing or recurrence.

## Discussion

A key finding of this review is not merely the magnitude of the observed pain reduction, but the scarcity and quality of the evidence base itself. Among 793 screened records, only seven RCTs (207 patients) met inclusion criteria; of eight registered trials, only two have been published. This reveals a critical evidence gap in the field of surgical decompression for DPN. Despite decades of clinical interest and numerous observational studies suggesting benefit, the RCT evidence base remains remarkably limited—and potentially biased. This finding in itself is a major contribution of our review: it highlights that current clinical practice is largely supported by low-certainty evidence, and underscores the urgent need for a systematic program of high-quality research.

According to a 2017 Agency for Healthcare Research and Quality report, no pharmacologic or nonpharmacologic treatment provides lasting relief for established DPN pain (29). Although NDSLL has been advocated as a beneficial treatment for DPN (16, 30, 31), its use in treating DPN has been controversial since it was reported in 1992 (11, 12). Medical specialists who emphasize medical management for treating DPN argue that the evidence does not sufficiently support using NDSLL in DPN treatment (11, 14, 32). Proponents argue that NDSLL can improve many aspects of patients with DPN, including pain, QOL, ulcer occurrence or recurrence, sensory, image, stability, and neuropathology (11, 27, 31, 33–37). Therefore, reviewing current high-quality clinical evidence is crucial. A systematic review in 2008 indicated that there were no single RCTs or other well-designed prospective studies confirming the applicability of NDSLL for diabetic symmetric distal neuropathy (15). The systematic review by Fadel et al. (38) included 6 RCTs and 16 observational studies. However, one of the 6 RCTs they included was a cohort study (39), and four were the results of different studies from LENS (23, 27–29). Other systematic reviews included a varying number of observational studies (16, 17, 19, 40). To our knowledge, this study is the only systematic review focused on evidence for RCTs, with four studies (7 articles) included.

The interpretation of pain outcomes in surgical trials is particularly challenging due to the well-documented placebo effect associated with surgical interventions. Meta-analyses of sham-controlled surgical trials have consistently demonstrated that approximately 30%–40% of the observed benefit in intervention groups can be attributed to nonspecific factors—including patient expectation, the therapeutic ritual of surgery, postoperative care, and natural history—rather than the specific anatomic procedure. In our review, only one of the seven included RCTs employed a sham surgery control. This represents a major methodological gap. Without sham controls, we cannot determine what proportion of the observed VAS reduction is specifically attributable to nerve decompression vs. placebo or expectation effects. Even the phenomenon of contralateral pain improvement observed in some within-patient comparison studies—while potentially reflecting neurophysiological cross-regulation (as we discuss above)—cannot be definitively distinguished from systemic placebo effects or reporting bias. Quantitatively, if one assumes that 30%–35% of the observed 2.77-point VAS reduction at 6 months is placebo-related, the specific treatment effect would be approximately 1.8–1.9 points—which still marginally exceeds the MCID threshold but with wide uncertainty. At 12 months, the lower bound of the 95% confidence interval (−0.29) already falls below the MCID threshold, suggesting that the placebo-adjusted effect may not be clinically meaningful in a substantial proportion of patients. This analysis underscores the urgent need for sham-controlled RCTs to definitively establish the specific efficacy of NDSLL.

This systematic review indicates that, compared with no surgery, NDSLL significantly reduces pain levels in patients with DPN at both POM6 and POM12 (Figures 3A,B), but there is a higher risk of wound-related complications (27/113). Moreover, compared with baseline, both the NDSLL and no surgery groups showed significant pain reduction at both POM6 and POM12 (Figures 3C–F). This phenomenon has raised concerns about the placebo effect (23, 27–29). However, pain relief was minimal in the no surgery group, particularly at POM12 (Figures 3D,F), indicating that the placebo effect was not significant. Moreover, to minimize placebo interference, Rozen et al. (24) conducted a 56-month follow-up. They found that, compared with the no-surgery group (*n* = 14), both the right-NDSLL group (*n* = 20) and the left-NDSLL group (*n* = 16) exhibited significantly greater pain reduction (right-NDSLL: mean difference, −7.65; 95% CI: −9.87 to −5.44; *p* < 0.0001; left-NDSLL: mean difference, −7.26; 95% CI: −9.60 to −4.91; *p* < 0.0001). In a 5-year follow-up study, Rinkel et al. found that although pain gradually increased after one year, mean VAS scores in the NDSLL group remained lower than those in the no-surgery group, with the difference being more pronounced in patients who underwent bilateral NDSLL (41). Therefore, the contralateral improvement observed after NDSLL surgery likely reflects active neurophysiological cross-regulation rather than a passive placebo effect (42). An explanation exists for the pain relief observed in the no surgery group. The spinothalamic tract, responsible for transmitting pain signals, exhibits partial ipsilateral and contralateral distribution during its ascending pathway. Therefore, pain signals perceived by both cortices decreased after NDSLL treatment on one lower limb. Moreover, the brain, brainstem and spinal cord have multiple descending paths which can extensively modulate and inhibit nociception (43). Bilateral descending inhibitory mechanisms targeting pain perception pathways remain active following NDSLL, potentially leading to bilateral pain relief.

RCTs showed that the pain score in the operated leg gradually increased after six months post-surgery (23, 28). The gradual increase in pain scores in the NDSLL group was likely due to pulling forces and the rigid texture of scar tissue, which could have caused re-entrapment. However, this does not explain why the pain scores in the no-surgery group showed similar changes. These studies showed that the placebo effect cannot be ignored. Since no sham surgery was performed in most of the trials, the placebo effect may have been further enhanced. The magnitude of the surgical placebo effect has been reported to be about the same as that of other placebo responses—about 35%—but with a longer duration (44). Rozen et al. (24) confirmed that, compared with sham surgery, pain did not improve significantly at all follow-up points within 12 months after surgery in the NDSLL group and significantly decreased at 56 months (*p* = 0.0002), and they also agreed that the placebo effect cannot be ignored. Overall, our systematic review and RCTs showed that NDSLL reduced pain in patients with DPN in the short term, but carries a high risk of bias (23, 24–26, 28). However, Rozen et al. (24) and Macaré van Maurik et al. (23, 28, 41) showed that NDSLL can significantly reduce pain in the long term compared with the control group. Our results and another RCT showed that NDSLL did not provide long-term pain relief compared to the control group (26). Moreover, pain was affected by blood glucose and body mass index (BMI), but only one RCT reported changes in blood glucose control and BMI after surgery (24).

The high risk of bias identified across the included RCTs, particularly the lack of participant and personnel blinding, the absence of sham surgery controls in 6/7 trials, incomplete outcome data, and selective reporting—has concrete implications for the interpretation of our pooled estimates. First, in surgical trials, the placebo effect is particularly substantial, with estimates suggesting that approximately 35% of the observed benefit in surgical interventions may be attributable to nonspecific factors such as patient expectation, the therapeutic ritual of surgery, and postoperative care, rather than the specific anatomic intervention itself. Without sham surgery, we cannot reliably distinguish the true decompression effect from this powerful placebo response. Consequently, the observed VAS reduction of 2.77 points at 6 months may include a sizable nonspecific component, and the specific treatment effect could be considerably smaller—potentially falling below the MCID threshold. Second, the lack of blinding introduces both performance bias (where knowledge of allocation may influence co-interventions and subjective symptom reporting) and detection bias (where unblinded outcome assessors may systematically favor the intervention group). Empirical evidence suggests that lack of blinding can inflate effect sizes by 20%–30% on average. Third, incomplete outcome data and selective reporting, as noted in our ROB assessment (Figure 2), further compound these issues by overrepresenting favorable results. Fourth, publication bias (75% non-publication rate of registered trials) suggests that the published literature is systematically skewed toward positive results. The unpublished trials are likely to have had null or negative findings; their omission from our meta-analysis likely inflates the pooled effect estimates. Collectively, these considerations suggest that the true treatment benefit of NDSLL, after accounting for these biases, may be substantially more modest than our pooled WMDs suggest, and may not consistently reach the MCID threshold across all patient populations.

Unlike other studies (24, 37, 41, 45), our results showed that NDSLL could not improve patients’ physical component summary scores, but other items were not evaluated due to incomplete data. Rozen et al. (24) indicated that the NDSLL group showed significant improvement in SF-36 physical component summary, bodily pain subscale, general health subscale, and vitality compared with the control group. However, the mental health component score did not differ between the decompression and control groups at POM 12. A prospective follow-up study by Fakkel et al. (37) also showed that NDSLL could improve QOL. However, two other RCTs reporting QOL showed no significant improvement after NDSLL (26, 28). Best et al. (26) attributed this to the small sample size or inadequacy of the NeuroQoL scale for this situation. A major limitation of another RCT was the within-patient comparison, which did not include observation of the control group (28). Therefore, comparing only the QOL changes before and after NDSLL may be unreasonable. This limitation could also help explain why our results showed no statistical difference. However, in their follow-up study, eight initial participants who underwent NDSLL of the contralateral leg had significantly lower pain scores and higher QOL compared with unilateral surgical patients five years after surgery (41). Therefore, the impact of NDSLL on QOL must be evaluated through double-blind, sham and parallel control, and long-term RCTs.

Several systematic reviews have reported that NDSLL could promote 2-PD recovery in the lower extremities and reduce the incidence of ulcers and amputations (16, 19, 38). Although two RCTS showed that NDSLL could improve 2-PD (25, 44), there was insufficient data to support these conclusions in our systematic review. Macaré van Maurik et al. (23) suggested that the number of patients with nonperception decreased from 32.7% to 19.9% after surgery (*p* = 0.025), but the number of patients with discriminating decreased from 17.0% to 4.1% (*p* = 0.006). In addition, we found no significant improvement in the preoperative and postoperative NCV of the tibial and common peroneal nerves. These results suggest that NDSLL does not prevent DPN progression. Liao et al. (46) reported that the cross-sectional area (CSA) was significantly decreased from baseline for the tibial (from 25.2 ± 3.8 to 18.4 ± 3.8 mm^2^) and common peroneal (from 21.0 ± 3.8 to 15.3 ± 3.2 mm^2^) nerves after NDSLL. Macaré van Maurik et al. (33) showed that CSA of the tibial nerve decreased 28 weeks after NDSLL, but there was no statistical difference compared with the baseline. However, no RCTs are available to support these conclusions.

A critical limitation of the current evidence base is the absence of demonstrable benefit on outcomes beyond pain. Our meta-analysis found no evidence that NDSLL improves quality of life, nerve conduction velocity, balance, ulcer healing, or sensory recovery. This is not merely an absence of statistical significance; it is an absence of evidence from adequately powered RCTs for outcomes that are central to patients’ functional status and long-term prognosis. The lack of improvement in NCV is particularly concerning, as it implies that decompression does not reverse the underlying neuropathic process—a finding consistent with the view that DPN is primarily a metabolic and microvascular disease, not simply a mechanical compression syndrome. The absence of evidence for ulcer prevention or healing similarly limits the intervention's value in diabetic foot care. The incomplete evidence for sensory recovery (due to unpoolable 2-PD data) leaves an important question unanswered. This pattern of findings suggests that NDSLL, if effective at all, may primarily serve as a symptom-palliative intervention rather than a disease-modifying therapy. This substantially constrains the clinical utility of NDSLL, particularly for patients whose primary concerns are ulcer prevention or functional decline rather than pain.

The interpretation of pain outcomes in surgical trials is particularly challenging due to the well-documented placebo effect associated with surgical interventions. Meta-analyses of sham-controlled surgical trials have consistently demonstrated that approximately 30%–40% of the observed benefit in intervention groups can be attributed to nonspecific factors, including patient expectation, the therapeutic ritual of surgery, postoperative care, and natural history, rather than the specific anatomic procedure. In our review, only one of the seven included RCTs employed a sham surgery control. This represents a major methodological gap. Without sham controls, we cannot determine what proportion of the observed VAS reduction is specifically attributable to nerve decompression vs. placebo or expectation effects. Even the phenomenon of contralateral pain improvement observed in some within-patient comparison studies, while potentially reflecting neurophysiological cross-regulation (as we discuss above), cannot be definitively distinguished from systemic placebo effects or reporting bias. Quantitatively, if one assumes that 30%–35% of the observed 2.77-point VAS reduction at 6 months is placebo-related, the specific treatment effect would be approximately 1.8–1.9 points, which still marginally exceeds the MCID threshold but with wide uncertainty. At 12 months, the lower bound of the 95% confidence interval (−0.29) already falls below the MCID threshold, suggesting that the placebo-adjusted effect may not be clinically meaningful in a substantial proportion of patients. This analysis underscores the urgent need for sham-controlled RCTs to definitively establish the specific efficacy of NDSLL.

Another controversial issue is the surgical indications. The theoretical basis for NDSLL is the double-crush. First, metabolic abnormalities lead to enlarged nerve trunks, and the epineural membrane causes the first entrapment. In areas like peri-articular fibro-osseous tunnels, failure to adapt to increased nerve size led to secondary entrapment (11). Therefore, some surgeons consider that a positive Tinel sign at a leg fibro-osseous tunnel site often indicates NDSLL (47). Other indications for NDSLL in DPN patients include symptoms of pain and/or a healed diabetic foot ulcer, failure of pain treatment, good glycemic control, a palpable foot pulse or ankle brachial pressure index >0.8, and minimal pedal edema (11). The indications of RCTs included in our systematic review were similar. The Tinel sign, while widely used in clinical practice, suffers from poor standardization, inter-observer variability, and limited sensitivity and specificity for detecting nerve entrapment in the setting of DPN. Several studies have demonstrated that a positive Tinel sign does not reliably predict surgical response or neurophysiological improvemen (35). Therefore, we do not recommend the Tinel sign as a primary or sole criterion for patient selection in clinical practice. Instead, the Tinel sign should be considered an area for further research and validation, particularly in conjunction with objective diagnostic tools such as high-resolution ultrasonography (measuring nerve cross-sectional area and retinaculum thickness) and electrophysiological criteria. Ultrasonography, in particular, shows promise as a more objective and reproducible tool (38), but its predictive value for surgical outcomes must be prospectively validated in sham-controlled RCTs before it can be recommended for routine clinical use. Until such validation is available, patient selection should be based on a comprehensive clinical evaluation, including careful consideration of the risk-benefit profile on an individual basis.

This review has several important limitations that affect the strength and reliability of our conclusions. Limited evidence base and statistical precision. The most fundamental limitation is the small number of eligible RCTs (*n* = 7) and modest cumulative sample size (*n* = 207), which directly impacts statistical power and the precision of our pooled estimates. As reflected in the wide confidence intervals (e.g., VAS at 12 months: 95% CI: −4.52 to −0.29), the true treatment effect may be substantially smaller than our point estimate suggests. Publication bias. The 75% non-publication rate of registered trials raises serious concerns that the published literature is systematically skewed toward positive results. Our pooled estimates may therefore represent upper-bound rather than unbiased effect sizes. A formal funnel plot was not constructed due to the small number of studies (<10), but the registered-to-published ratio provides compelling qualitative evidence of publication bias. High risk of bias. Most included RCTs were at high risk of bias regarding blinding, sham controls, incomplete outcome data, and selective reporting. These flaws likely inflate observed treatment effects. As discussed above, the lack of sham surgery (in 6/7 trials) and absence of blinding mean we cannot differentiate specific from nonspecific effects. Methodological heterogeneity. Considerable heterogeneity was observed in patient selection criteria, baseline pain severity, Tinel sign use, follow-up duration, and outcome assessment tools. This heterogeneity—which could not be explored via subgroup analyses due to study number limitations—reduces generalizability and precision. Incomplete outcome reporting for secondary outcomes. Data on 2-PD and TCSS could not be pooled due to incomplete reporting. This may itself reflect selective reporting and prevents robust conclusions about sensory and neurological outcomes. Overall certainty of the evidence. Considering the cumulative impact of the limitations described above, the current body of evidence provides low certainty regarding the true efficacy of NDSLL for pain reduction. For secondary outcomes (QOL, NCV, balance, ulcer healing, and sensation), the evidence provides very low certainty due to sparse data, incomplete reporting, and inconsistent measurement tools. Therefore, our findings should be interpreted as preliminary and hypothesis-generating, providing a foundation for designing future definitive trials rather than offering conclusive evidence to change clinical practice.

Although the question of the surgical indications for NDSLL is important, our study showed that with the available literature and the heterogeneity in definitions, it is impossible to answer this question. Objective indicators to replace the Tinel sign need to be found. Large studies targeting specific types of patients with DPN for NDSLL might help get closer to an answer. Moreover, although NDSLL was associated with reduced pain, its effects must be evaluated through double-blind, sham and parallel control, and long-term RCTs.

Our study suggests that NDSLL may reduce pain in patients with painful DPN at 6 and 12 months postoperatively. However, this evidence is derived from a small number of high-bias RCTs with substantial publication bias, considerable methodological heterogeneity, a notable complication rate (∼24%), and no demonstrable improvement in QOL, NCV, balance, ulcer healing, or sensation. The true treatment effect may be substantially smaller than pooled estimates suggest, and the risk-benefit balance is precarious. Therefore, the current evidence does not justify widespread adoption of NDSLL; rather, it underscores the urgent need for well-designed, sham-controlled, multicenter RCTs with standardized outcome measures and mandatory results disclosure. NDSLL should remain an investigational procedure until such evidence becomes available.

## Data Availability

The original contributions presented in the study are included in the article/Supplementary Material, further inquiries can be directed to the corresponding authors.
