# Supplementary material for: Nerve decompressive surgery of the lower limbs for diabetic peripheral neuropathy: a systematic review and meta-analysis
Source: Front Pain Res (Lausanne). 2026 Jul 30;7:1879398. doi: 10.3389/fpain.2026.1879398 (PMC13469247; doi:10.3389/fpain.2026.1879398)
Supplement: Supplementary file 1 [file Table1.docx]

**Supplementary Materials**

**Table of Contents**

**Appendix 1: Search Strategies**

**Appendix 1: The inclusion and exclusion criteria**

**Appendix 2: List of important excluded studies and protocols**

**Appendix 3: The inclusion and exclusion criteria of included studies**

**Appendix 4: Detailed characteristics of included studies**

**Appendix 5: Forest plots for common peroneal nerve conduction velocity**

**Appendix 1: Search Strategies**

Database: PubMed

Search Strategy:

1. exp Diabetes Mellitus / (524522)
2. diabet*.mp. (815655)
3. 1 OR 2 (877340)
4. exp Peripheral Nervous System Diseases / (169759)
5. (neuropath* or polyneuropath* or mononeuropath* or “DPN”).mp. (179626)
6. 4 OR 5 (289640)
7. 3 AND 6 (34319)
8. exp diabetic neuropathies / (27,538)
9. 7 OR 8 (43426)
10. exp Decompression, Surgical / (36,580)
11. decompress*.mp. (57505)
12. 10 OR 11 (76200)
13. 9 AND 12 (334)

Database: Embase

Search Strategy:

1. exp Diabetes Mellitus / (1330457)
2. diabet*.mp. (1243848)
3. 1 OR 2 (1543037)
4. exp Peripheral Nervous System Diseases / (99282)
5. (neuropath* or polyneuropath* or mononeuropath* or “DPN”).mp. (256940)
6. 4 OR 5 (301218)
7. 3 AND 6 (58992)
8. exp diabetic neuropathies / (31279)
9. 7 OR 8 (58992)
10. exp Decompression, Surgical / (1830)
11. decompress*.mp. (67483)
12. 10 OR 11 (111351)
13. 9 AND 12 (412)

Database: the Cochrane Library

Search Strategy:

14. exp Diabetes Mellitus / (45767)
15. diabet*.mp. (121515)
16. 1 OR 2 (121877)
17. exp Peripheral Nervous System Diseases / (8155)
18. (neuropath* or polyneuropath* or mononeuropath* or “DPN”).mp. (18758)
19. 4 OR 5 (22469)
20. 3 AND 6 (5834)
21. exp diabetic neuropathies / (2307)
22. 7 OR 8 (7004)
23. exp Decompression, Surgical / (1830)
24. decompress*.mp. (4315)
25. 10 OR 11 (5174)
26. 9 AND 12 (47)

**Appendix 2: The inclusion and exclusion criteria**

| Inclusion criteria | Exclusion criteria |
| --- | --- |
| 1. People with either type 1 or 2 diabetes mellitus and established DPN；  2. Age ≥ 18 years, and regardless of gender or setting;  3.RCTs and quasi-RCTs that compare NDSLL with a non-surgical treatment, sham surgery or no treatment  4. No language restriction;  5. Follow-up period of greater than 3 months;  6. Primary outcome was change in pain measured by the VAS or other scale;  7. Secondary outcomes included any validated assessments of nerve function, QOL, surgical complications, and diabetic foot ulcers. | 1. Patients with other types of peripheral neuropathy, such as cerebral infarction, Guillain-Barre syndrome, severe venous vascular disease, cervical spondylosis, and lumbar lesion;  2. Preclinic studies, case reports/case series, cost-effectiveness analysis, cohort study, retrospective analysis, consensus papers, Retrospective analysis conference proceedings, abstracts, letters, reviews, anatomical/cadaveric studies, systematic and reviews meta-analysis |

DPN, diabetic peripheral neuropathy; RCTs, randomized controlled trials; NDSLL, nerve decompressive surgery of lower limbs; VAS, visual analogue scale; QOL, quality of life.

**Appendix 3: List of important excluded studies and protocols**

| Study ID | Title | Excluded cause |
| --- | --- | --- |
| Nickerson 2024 | Objective Evidence that Nerve Decompression Surgery Reduces Neuropathic DFU Recurrence Risk to Less Than 5 | Review |
| Fakkel 2022 | Does Lower Extremity Nerve Decompression Surgery Improve Quality of Life? | Case series |
| ChiCTR2200062283 | Peripheral nerve microdecompression combined with recombinant human acidic fibroblast growth factor in the treatment of diabetic peripheral neuropathy | No raw data |
| ChiCTR2200060034 | Clinical study of modified neurolysis in the treatment of lower extremity diabetic peripheral neuropathy | No raw data |
| Uemura 2021 | A Minimally Invasive Full Endoscopic Approach to Tibial Nerve Neurolysis in Diabetic Foot Neuropathy: An Alternative to Open Procedures | Case series |
| Rinkel 2021 | Cost-Effectiveness of Lower Extremity Nerve Decompression Surgery in the Prevention of Ulcers and Amputations: A Markov Analysis | Cost-effectiveness analysis |
| ChiCTR2100050049 | Peripheral nerve decompression for painful diabetic peripheral neuropathy in lower extremities: a single-center, open-label randomized controlled trial | No raw data |
| ChiCTR2100044552 | The effect of neurolysis combined with rat nerve growth factor on peripheral neuropathy of diabetic foot | No raw data |
| ChiCTR2100053581 | Application and efficacy analysis of NTFs functional bioscaffold in PDPN nerve decompression | No raw data |
| Yamasaki 2020 | Acute Improvement in Intraoperative EMG During Common Fibular Nerve Decompression in Patients with Symptomatic Diabetic Sensorimotor Peripheral Neuropathy: EMG and Clinical Attribute Interrelations | Retrospective analysis |
| Agarwal 2020 | Tarsal tunnel release restores sensations in sole for diabetic sensorimotor polyneuropathy | Case series |
| Sarmiento 2019 | Tibial nerve decompression for the prevention of the diabetic foot: a cost-utility analysis using Markov model simulations | Cost-effectiveness analysis |
| Rinkel 2018 | Optimization of Surgical Outcome in Lower Extremity Nerve Decompression Surgery | Case series |
| Anderson 2017 | Acute Improvement in Intraoperative EMG Following Common Fibular Nerve Decompression in Patients with Symptomatic Diabetic Sensorimotor Peripheral Neuropathy: 1. EMG Results | Retrospective study |
| Yang 2016 | Pain Relief and Health-Related Quality-of-Life Improvement After Microsurgical Decompression of Entrapped Peripheral Nerves in Patients With Painful Diabetic Peripheral Neuropathy | Case series |
| Trignano 2016 | Evaluation of peripheral microcirculation improvement of foot after tarsal tunnel release in diabetic patients by transcutaneous oximetry | Case series |
| Tekin 2015 | The ultrasonographic evaluation of hemodynamic changes in patients with diabetic polyneuropathy after tarsal tunnel decompression | Retrospective study |
| Zhong 2014 | Impact of diabetes mellitus duration on effect of lower extremity nerve decompression in 1,526 diabetic peripheral neuropathy patients | Cohort study |
| Nickerson 2014 | Nerve decompression after diabetic foot ulceration may protect against recurrence: a 3-year controlled, prospective analysis | Cohort study |
| Macaré van Maurik 2014 | Ultrasound findings after surgical decompression of the tarsal tunnel in patients with painful diabetic polyneuropathy: a prospective randomized study | Cohort study |
| Zhang 2013 | Evaluation of the clinical efficacy of multiple lower-extremity nerve decompression in diabetic peripheral neuropathy | Cohort study |
| Zhang 2013 | Evaluation of the clinical efficacy of multiple lower extremity nerve decompression in diabetic peripheral neuropathy | Cohort study |
| Nickerson 2013 | Low long-term risk of foot ulcer recurrence after nerve decompression in a diabetes neuropathy cohort | Case series |
| NCT01762085 | Nerve Decompression for Ulcer Recurrence Avoidance (DURA) | No raw data |
| Dellon 2012 | A positive Tinel sign as predictor of pain relief or sensory recovery after decompression of chronic tibial nerve compression in patients with diabetic neuropathy | Case series |
| NCT01006915 | Surgical Decompression for Diabetic Neuropathy in the Foot | Completed. Included in this review |
| NCT00703209 | Study on the Role of Decompression of Lower Extremity Nerves for the Treatment of Patients With Symptomatic Diabetic Neuropathy With Chronic Nerve Compression | Completed. Included in this review |
| Karagoz 2008 | Early and late results of nerve decompression procedures in diabetic neuropathy: A series from Turkiye | Case series |
| Dellon 2008 | Reversal of toe clawing in the patient with neuropathy by neurolysis of the distal tibial nerve | Case series |
| Nelson 2007 | The 36-Item Short-Form Health Survey outcome evaluation for multiple lower-extremity nerve decompressions in diabetic peripheral neuropathy a pilot study | Case series |
| Cornblath 2007 | Surgical decompression for diabetic sensorimotor polyneuropathy | Comments |
| Ducic 2006 | Relationship between peripheral nerve decompression and gain of pedal sensibility and balance in patients with peripheral neuropathy | Case series |
| Valdivia 2005 | Surgical treatment of peripheral neuropathy: outcomes from 100 consecutive decompressions | Case series |
| Rader 2005 | Surgical decompression in lower-extremity diabetic peripheral neuropathy | Case series |
| Wieman 1995 | Treatment of hyperesthetic neuropathic pain in diabetics. Decompression of the tarsal tunnel | Case series |

**Appendix 4: The inclusion and exclusion criteria of included studies**

| Study ID | Inclusion criteria | Exclusion criteria |
| --- | --- | --- |
| Rozen 2024 | 1. Type 1 or 2 diabetes mellitus; all genders and all races;  2. Ages 18-80 years;  3. Symptoms consistent with peripheral neuropathy: numbness, pain and paresthesia, numbness & pain, with or without a motor component;  4. Bilateral Symptoms;  5. Neurologic history, examination, symptoms and signs consistent with symmetrical sensory or sensorimotor diabetic polyneuropathy;  6. Patients who have been treated non surgically for at least a year prior to entering the study and continue to have symptoms and / or have stopped pharmaceutical intervention due to treatment failure or experience sided effects prior to a one-year perioda;  7. Willingness to follow with physician treatment & instructions;  8. Positive Tinel sign (tenderness upon percussion over the region of supposed nerve compression) on examination over the fibular neck, tarsal tunnel, or both;  9. Nerve conduction studies are consistent with symmetrical peripheral neuropathy, including reduced amplitude of the sural sensory response. | 1. History of foot ulcers;  2. Symptoms of radiculopathy or sciatic type pain;  3. Weight > 136 kg;  4. Previous amputations involving lower extremities;  5. Cardiac or renal pedal edema;  6. Medical contra-indications for surgery;  7. Inadequate glycemic control (HbA1c (%) > 12);  8. Charcot foot deformity;  9. Substance dependency or History of noncompliance;  10. Inadequate circulation to feet and/or no palpable pulses (Ankle Brachial Index < 0.8);  11. Pregnancy. |
| Ma 2023 | 1. Type 2 diabetes mellitus;  2. Patients presented with spontaneous neuralgia below the knees and on the bottom of the foot with sensory disturbance;  3. Electromyography (EMG) confirmed peripheral neuropathy in the lower limbs;  4. Neural Tinel’s sign was positive, and patients had no severe edema of the feet. | Peripheral neuropathy in the lower limbs caused by cervical and lumbar spine lesions, infectious polyneuritis, chronic alcoholism, drug poisoning, thyroid disease, tumors, cerebrovascular sequelae, and peripheral vascular disease were excluded in all patients. |
| Best 2019 | 1. Age ≥ 18 years;  2. Diabetes mellitus type 1 or 2 in accordance with the Canadian Diabetes Association 2003 Clinical Practice Guidelines; 3. Good diabetic control with hemoglobin A1C (HbA1c) less than 8.0% (64 mmol/mol);  4. Experiencing symptoms of paresthesia (including burning pain) or numbness present symmetrically in both feet, determined to be on a peripheral nerve basis clinically and using nerve conduction studies interpreted by a consultant neurologist;  5. Average daily pain ≥ 5 on the 10-point McGill Pain Questionnaire visual analogue item;  6. A total neuropathy score (TNS) ≥ 2. | 1. Other etiology diabetes mellitus or neuropathy;  2. Symptomatic lumbosacral spine or lower extremity vascular disease;  3. Previous foot ulceration or amputation; HbA1c ≥ 8.1% (65 mmol/mol);  4. Significant ankle edema, venous stasis, morbid obesity, or previous surgery/injury which would be incompatible with appropriate wound healing;  5. Adults lacking capacity to consent, pregnant women, prisoners, non-English speakers and those unwilling or unable to participate in the full study follow-up. |
| Macaré van Maurik 2014, 2015 | 1. A positive Tinel sign at one or more of the following locations: the posterior tibial, superficial, or common peroneal nerve; 2. Ankle-brachial index value between 0.8 and 1.15;  3. A toe-brachial index of 0.7 or greater;  4. Palpable pulsations in the posterior tibial artery and dorsal pedal artery;  5. Pain score measured with visual analogue scale >2;  6. Diabetes mellitus Type 1 or Type 2;  7. Age 18-90 years;  8. Painful bilateral DPN, diagnosed using the Diabetic Neuropathy Score and Diabetic Neuropathy Examination. | 1. Body mass index (BMI) > 35 kg/m^2^;  2. General condition unsuitable for surgery;  3. History of ankle fractures or amputations proximal to the Lisfranc joint;  4. Ulcer on the foot;  5. Sufficient effect of pain medication (visual analogue scale score, 0 to 1);  6. Other causes for neuropathy (e.g., human immunodeficiency virus, chemotherapy);  7. Indications for and history of nerve compression at additional sites (e.g., hernia or nerve root compression);  8. Impossibility to stand barefoot for 30 seconds. |

**Appendix 5: Detailed characteristics of included studies**

| Study ID | Year | Country | Status | Intervention (Sample) | Control (Sample) | M/F | | BMI | | Duration of Pain (months) | | Type 2 diabetes, n (%) | | DPN duration (years) | | HbA1c (mean ±SD) | |
| --- | --- | --- | --- | --- | --- | --- | --- | --- | --- | --- | --- | --- | --- | --- | --- | --- | --- |
|  |  |  |  |  |  | Intervention | Control | Intervention | Control | Intervention | Control | Intervention | Control | Intervention | Control | Intervention | Control |
| Rozen 2024 | 2024 | USA | developed | NDSLL (40) | A: sham (40). B: No surgery (37) | 19/21 | A: 19/21  B: 22/15 | 32.94 ± 7.71 | A: 32.94 ± 7.71  B: 31.83 ± 7.33 | unknown | unknown | 38 (95) | A: 38 (95)  B: 36 (97.3) | 3.00 ± 2.00 | 4 ± 6.5 | 7.98 ± 1.98 (%) | 8.43 ± 3.15 (%) |
| Ma 2023 | 2023 | China | developing | NDSLL (23) | A: No surgery (23). B: NDSLL+ nerve conduit (23) | 12/11 | A: 12/11  B: 13/10 | NR | NR | 16.3 ± 8.6 | A：13.1 ± 5.4  B: 16.3 ± 8.6 | 23 (100) | A: 23 (100)  B: 23 (100) | NR | NR | NR | NR |
| Best 2019 | 2019 | Canada | developed | NDSLL (12) | No surgery (10) | 6/6 | 4/6 | NR | NR | NR | NR | 12 (100) | 9 (90) | NR | NR | 6.8 ± ? (%) | 6.4 ± ? (%) |
| Macaré van Maurik 2014, 2015 | 2014, 2015 | Netherlands | developed | NDSLL (38) | No surgery (38) | 22/16 | 22/16 | 29.0 ± 4.2 | 29.0 ± 4.2 | NR | NR | 30 (78.9) | 30 (78.9) | NR | NR | 57.3 ± 13.8 (mmol/mol ) | 57.3 ± 13.8 (mmol/mol ) |

M/F: male and female; BMI: body mass index; DPN: diabetic peripheral neuropathy; HbA1c: glycated hemoglobin A1c

**Appendix 6: Forest plots for common peroneal nerve conduction velocity**

Pooled analysis of common peroneal NCV. A, Pooled analysis of NDSLL versus no-surgery in common peroneal NCV. B, Pooled analysis of preoperative versus postoperative posterior common peroneal NCV in the NDSLL group. C, Pooled analysis of preoperative versus postoperative common peroneal NCV in the no-surgery group. NCV, nerve conduction velocity; NDSLL, nerve decompressive surgery of lower limbs
